# Validation of three deformable image registration algorithms for the thorax

**DOI:** 10.1120/jacmp.v14i1.3834

**Published:** 2013-01-07

**Authors:** Kujtim Latifi, Geoffrey Zhang, Marnix Stawicki, Wouter van Elmpt, Andre Dekker, Kenneth Forster

**Affiliations:** ^1^ Division of Radiation Oncology H. Lee Moffitt Cancer Center Tampa FL USA; ^2^ Department of Radiation Oncology (MAASTRO) Maastricht University Medical Centre NL‐6229 ET Maastricht The Netherlands; ^3^ Department of Physics University of South Florida Tampa FL USA; ^4^ Department of Radiation Oncology The Mitchell Cancer Institute at University of South Alabama Mobile AL USA

**Keywords:** DIR, image registration, deformable, validation, 4D CT

## Abstract

Deformable image registration (DIR) has been proposed for lung ventilation calculation using 4D CT. Spatial accuracy of DIR can be evaluated using expert landmark correspondences. Additionally, image differences between the deformed and the target images give a degree of accuracy of DIR algorithms for the same image modality registration. DIR of the normal end‐expiration (50%), end‐ inspiration (0%), midexpiration (30%), and midinspiration image (70%) phases of the 4D CT images was used to correlate the voxels between the respiratory phases. Three DIR algorithms, optical flow (OF), diffeomorphic morphons (DM), and diffeomorphic demons (DD) were validated using a 4D thorax model, consisting of a 4D CT image dataset, along with associated landmarks delineated by a radiologist. Image differences between the deformed and the target images were used to evaluate the degree of registration accuracy of the three DIR algorithms. In the validation of the DIR algorithms, the average target registration error (TRE) for normal end‐expiration‐to‐end‐inspiration registration with one standard deviation (SD) for the DIR algorithms was 1.6±0.9mm (maximum 3.1 mm) for OF, 1.4±0.6mm (maximum 3.3 mm) for DM, and 1.4±0.7mm (maximum 3.3 mm) for DD, indicating registration errors were within two voxels. As a reference, the median value of TRE between 0 and 50% phases with rigid registration only was 5.0 mm with one SD of 2.5 mm and the maximum value of 12.0 mm. For the OF algorithm, 81% of voxels were within a difference of 50 HU, and 93% of the voxels were within 100 HU. For the DM algorithm, 69% of voxels were within 50 HU, and 87% within 100 HU. For the DD algorithm, 71% of the voxels were within 50 HU, and 87% within a difference of 100 HU. These data suggest that the three DIR methods perform accurate registrations in the thorax region. The mean TRE for all three DIR methods was less than two voxels suggesting that the registration performed by all methods are equally accurate in the thorax.

PACS number:89.90

## I. INTRODUCTION

In image registration, images are aligned by establishing a correspondence between features in both images. We can categorize these algorithms into two categories: rigid registration and deformable image registration (DIR). Rigid registration simply aligns the images, whereas DIR can take into account complex changes such as deformation due to breathing, patient weight loss, or tumor shrinkage. The purpose of DIR is to find a transformation from one image set to another, such that the differences between the deformed and the target image sets are minimized by providing a voxel‐to‐voxel deformation matrix. DIR has been studied since the early 1980s. For many years, neurosciences and neurosurgery have been the driving force for developing various deformation techniques.^(^ ^1^ ^,^ ^2^ ^)^ A more recent application of DIR is its use in the calculation of ventilation from four‐dimensional computed tomography (4D CT), which is also our motivation for validating the DIR methods.^(^ ^3^ ^–^ ^11^ ^)^

An application of DIR as exemplified in this paper is image registration of two images from two different times during a breathing cycle (4D CT). DIR finds a mapping between a voxel in one phase of the image (e.g., end expiration) to a corresponding voxel in the second phase (e.g., end inspiration).

A variety of DIR algorithms have been implemented. While the goal of these algorithms is the same, the algorithms are based on aligning different image features, how they measure similarity, and what type of deformations they allow. In this paper, DIR between different phases (0%, 30%, 50%, and 70%) of the breathing cycle was evaluated by three different DIR algorithms, optical flow (OF),^(^ ^12^ ^–^ ^15^ ^)^ diffeomorphic demons (DD),^(^ ^16^ ^–^ ^18^ ^)^ and diffeomorphic morphons (DM).^(^ ^19^ ^)^ A more thorough description of these algorithms is given in the corresponding Methods section.

For all DIR algorithms, it is highly desirable to provide an estimate of the accuracy of their registration for the desired application.^(^ ^20^ ^–^ ^26^ ^)^ The algorithms investigated in this paper were all evaluated using an anatomical landmark‐based model, point‐validated pixel‐based breathing model (POPI),^(^ ^23^ ^,^ ^24^ ^)^ and the image differences between the deformed and the target lung images. The model consists of anatomical landmarks in the lung chosen by expert radiologists. The intention of the model is to have landmarks that are spread uniformly and correspond to anatomical features such as the carina, calcified nodules, division branch of the pulmonary artery, and apical pulmonary vein of the upper lobe and various bifurcations of smaller structures that could be uniquely identified in each image set by four different medical experts. A more detailed procedure for obtaining the landmarks is given by Sarrut et al.^(^ ^22^ ^)^

The POPI model is a useful tool that has been made available to researchers, which can be used to estimate the deformation accuracy in the lung. This paper evaluates the accuracy of OF, DD, and DM DIR algorithms that are used for registration of the 4D CT image sets for the application of accurate DIR for the lung.

## II. MATERIALS AND METHODS

### A. deformable image registration algorithms

Three different registration algorithms, OF,^(^ ^12^ ^–^ ^14^ ^,^ ^27^) DM,^(^ ^25^ ^,^ ^28^ ^)^ and DD(^18,^, ^25,^, ^29^) were used to deform the end‐expiration image (50%) to the end‐inspiration (0%), midexpiration (30%), and midinspiration image (70%).

#### A.1 Optical flow

OF finds the voxel correspondence by computing a displacement field describing the apparent motion represented by the two image sets by matching the intensity gradients.^(^ ^13^ ^–^ ^15^ ^)^ OF assumes that Houndsfield Unit (HU) values remain constant for the corresponding voxels. HU of a voxel at time t is equal to the HU at a later time t+δt, that is:

(1) $$I(\vec{x},t)\approx I(\vec{x}+\delta \vec{x},t+\delta t),$$

where $I(\bar{x},t)$ is the HU value at time *t*. Applying Taylor series expansion of the terms on the right‐hand side of Eq. (1) and ignoring terms higher than the first derivative, we obtain the optical flow constraint equation:

(2) $$\left(\vec{\nabla }I\cdot \vec{v}+\frac{\partial I}{\partial t}\right)(\vec{x},t)=0,$$

where $\vec{v}=\frac{\partial \vec{x}}{\partial t}$ is the velocity and it consists of the displacement between a point in the first image, $I(\bar{x},t)$, and the corresponding point in the second image $I(\vec{x}+\delta \vec{x},t+\delta t)$ divided by the time between the two images, δt. A velocity constraint, which requires that nearby points move in a similar manner to each point, is introduced in order to obtain a single solution from Eq. (2) for each voxel. Using calculus of variations, an iterative approach was applied to solve for the displacement vector field in the implemented OF computer program.^(^ ^13^ ^,^ ^27^ ^)^

#### A.2 Diffeomorphic demons

**Figure 1:**
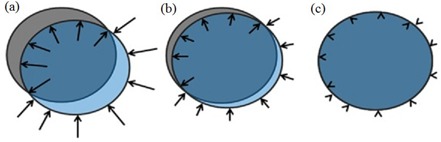
Illustration of the demons forces (a) with target image (T) in gray and moving image (M) in transparent blue; the demons indicated by vector arrows warp the image by applying a force in the direction of the image gradient. In (b), there is a better overlap between the images and, as a result, the corresponding force is reduced, indicated by shorter vectors. In (c), the images overlap and there is no applied force by the demons because there is no difference in the gradient.

Maxwell's demon was adapted by Thirion(^17^) to a diffusion‐based algorithm as a fast technique for deformable registration of medical images. In DIR using DD algorithms, the matching is based on HU values between image sets. The goal of DD, similar to the OF algorithm, is the minimization of HU differences between the voxels in the moving image (M), which is the image to be deformed, and the corresponding voxels in the target image (T), which is the image that M is deformed to match. As illustrated in Fig. 1, demons forces are applied on the moving image until there is an overlap in HU between the two. The difference in HU between the two images (M ‐ T) determines the applied force and its direction. When the difference in HU between the two is greater than zero, M moves in the direction of $\bar{\nabla }T$; however, when the difference is less than zero, M moves against $\bar{\nabla }T$. The demons stop exerting force when the images overlap completely. The optical flow equation was used to calculate the force applied by the demons, but is renormalized because of the effects of small image intensity gradients.

It was demonstrated by Pennec et al.^(^ ^16^ ^)^ that this method resembles an optimization of a second order gradient descent of the sum of the squared intensity differences (SSD). This indicates that demons would function well in single modality registration, where SSD gradient descent is also appropriate.

Demons algorithm is adapted to include the gradients of the moving and target images. This optimization makes demons more efficient, and is the method that is implemented for our demons registrations. Additionally, to create physical displacement fields, the demons algorithm is updated to make it diffeomorphic.^(^ ^18^ ^)^ A diffeomorphism is a map between manifolds, which is differentiable, and its inverse is differentiable, as well.^(^ ^18^ ^)^ Organs can be compressed and deformed and undergo invertible spatial transformation.

The deformation field produced by DD is smoothed by a Gaussian filter, and iteratively used to transform the moving image, and register on to the static image. The displacement vectors during deformation are calculated independently for every voxel. Due to the image noise, the displacement field may not be smooth, so regularization is applied to get a smoother deformation field.

#### A.3 Diffeomorphic morphons

The DM algorithm is based on matching of edges and lines.^(^ ^25^ ^)^ The difference between this algorithm and OF and DD is that the computation of the field is based on the local phase difference rather than the intensity difference between images. The phase difference between periodic signals with the same frequency allows the estimation of the spatial difference between these signals. The morphon iteratively deforms a moving image into a target image by morphing the moving image. The process can be divided into three parts: estimation of displacement, accumulation of the deformation field, and deformation.

The estimation of displacement has the aim to find the ways and indications on how to deform the moving image into the target image. Estimation of displacement is based on quadrature phase difference. The accumulation of the deformation field uses the estimate of the displacement to update the deformation field. Finally, the deformation morphs the moving image to the target image according to the accumulated deformation field. These steps are done iteratively, as long as the displacement estimates indicate further morphing to be done.^(^ ^19^ ^,^ ^25^ ^)^

Quadrature phase difference is a method used to estimate local displacement between two images. The advantage of this method over other methods, such as the ones based on gradient and polynomial expansion, is its invariance to image intensity and weak gradients.^(^ ^19^ ^)^ Edges between bright and dark areas have one phase, dark lines have one phase, and lines on dark background have a phase as well as bright patches. The transition as we move from one phase to another is continuous. Therefore, the difference in local phase between the moving and target images is a good measure of how much the moving image has to move to fit the target image. The local displacement is a function of the local phase along its associated direction. To estimate the local displacement a least square estimate is used.

The moving image is deformed based on the accumulated field and then it is compared to the target image in order to estimate a displacement field for the current iteration being performed. The updated field is formed by combining the accumulated field and the displacement field from the current iteration. After acquiring the update field as well as the certainty from the field, a weighted accumulation is used to determine the accumulated displacement field. Additionally, estimation of the displacement vectors is combined with a measure of the estimation confidence, which results in a certainty map. In order to prevent folding, the morphons algorithm is optimized to become diffeomorphic. As in the DD algorithm, the deformation field produced by the DM is smoothed by a Gaussian filter, and iteratively used to transform the moving image and register to the static image.

#### A.4 Implementation

All three DIR algorithms are implemented in an iterative and multiscale scheme. A lower resolution deformation is first approximated, then used for the next level of deformations with a higher resolution of the images to be registered. This process continues until the deformation has been approximated at the highest resolution.^(^ ^14^ ^,^ ^18^ ^,^ ^19^ ^,^ ^22^ ^)^

OF is implemented using C++ with four scales of resolution, and 98 iterations at each scale, which was determined to be the optimal number of iterations for OF. DD and DM were implemented using MATLAB (The MathWorks, Natick, MA). The settings were the same as the ones described in Janssens et al.^(^ ^25^ ^)^ that is using eight scales, with a minimum of 10 and a maximum of 20 iterations at each scale, and a Gaussian smoothing with a standard deviation σ=2. The iterative process was stopped if the changes measured in terms of the sum of the squared differences (SSD) were less than 0.01%.^(^ ^25^ ^)^

### B. Validation of deformable image registration algorithms

All three DIR algorithms were evaluated using the POPI model. The POPI model consists of a typical clinical 4D CT image dataset of a lung with the images binned into 10 phases along with 41 associated anatomical landmarks. The 4D CT images were acquired using a Phillips Brilliance Big Bore CT (Philips Healthcare, Andover, MA), and an air bellows belt with a pressure transducer was used to convert the pressure waveform into respiratory phase information, which was used for the 4D image phase tagging.^(^ ^24^ ^,^ ^30^ ^)^ The landmarks are anatomically homologous points that were manually delineated by radiologists at all 10 phases of the original 4D CT image sets.^(^ ^24^ ^)^ They are based on anatomical features that correspond to various locations in the lung such as the carina, calcified nodules, culmen‐lingula junction, division branch of pulmonary artery, and apical pulmonary vein of the upper lobe. The CT image sets consisted of 512×512×141 voxels with voxel dimensions of $0.97\times 0.97\times 2\;{\text{mm}}^{2}$ (2 mm slice thickness). The phases used for the evaluation of the DIR algorithms were normal end inspiration (0% phase), midexpiration (30% phase, the phase that lies between the end inspiration and end expiration), normal end expiration (50% phase), and the 70% phase which is midinspiration or the phase that lies between the end expiration and end inspiration. For all the methods, the original datasets were used for this validation without any changes to the images.

Using deformable registration, one phase is registered to the second phase (e.g., end‐expiration‐to‐end‐inspiration phase). This generates deformation vector fields (DVFs) that approximate the anatomical displacements voxel‐by‐voxel. The vector fields point from the target positions to the new displaced positions of the anatomical features. For this study the 0%, 30%, and 70% phases were each registered to the 50% phase.

The resulting deformation fields were used to calculate the new location of the landmarks on the end‐expiration phase. Then, the positions of the calculated landmarks were compared to the inspiration phase landmarks, as determined by the radiologists. TRE was then assessed by calculating the displacement between the calculated and the anatomically determined landmarks for each of the radiologist identified points ($\text{TRE}=X_{a}$ ‐ $X_{b}$, where $X_{a}$ is the anatomical position of the landmark and $X_{b}$ is the calculated position of the landmark). This displacement was used as the metric to assess the accuracy of the registration algorithms. For comparison purposes, the difference in position between landmarks with rigid registration (RIG) only was calculated. That difference is defined as the position of the landmarks in the inspiration phase minus the position of the landmarks in the expiration phase.

Additionally, the DIR algorithms were evaluated on a voxel‐to‐voxel basis by calculating the HU differences between the deformed image and the static image. Inspecting image differences is convenient and may provide qualitative evaluation of the accuracy of the DIR. After an accurate deformation, the HU differences between the deformed and static images should be small. However, in reality, small misalignments can have a big effect and the differences can be large, especially near interfaces. The difference in HU between the deformed and target images is used as an indicator of the accuracy of the registration. A region of interest (ROI) was drawn to include only the voxels inside the lung and exclude the rest of the image. For comparison, the differences between the moving and the target images were calculated using RIG only.

## III. RESULTS

Only 39 of 41 landmarks in the POPI model were used. Two of the landmarks were intentionally not used for the evaluation because one of them was outside the area of interest and the other one corresponded to a 4D CT artifact.

**Figure 2:**
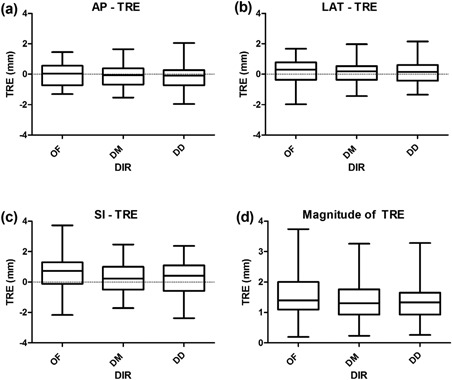
Box plot graphical representation of the ANOVA results for: (a) AP, (b) LAT, (c) SI, and (d) magnitude TRE for the OF, DM, and DD DIR.

Table 1 shows a summary of the statistics of target registration errors for the OF, DM, and DD algorithms for registration between the end‐expiration and end‐inspiration (50% and 0%) phases, 50% and 30%, 50% and 70%, respectively. The data show that the registration errors were within two voxels. One‐way analysis of variance (ANOVA) was used to assess the differences between the three DIR algorithms. The differences between the TREs using OF, DM, and DD were not significant, p‐value of 0.37. Box plot graphical representations of the ANOVA results are shown in Fig. 2.

**Table 1:** Summary of the magnitude of the target registration errors for the three DIR algorithms and the distances between landmarks with RIG only: (upper) for 50%–0%, (middle) for 50%–30%, (lower) for 50%–70%.

		*50%–0%*		
*Phases*	*OF*	*DM*	*DD*	*RIG*
Median (mm)	1.5	1.3	1.3	5.0
SD (mm)	0.9	0.6	0.7	2.5
Max (mm)	3.7	3.3	3.3	12.0
Min (mm)	0.2	0.2	0.3	2.5
		*50%–30%*		
*Phases*	*OF*	*DM*	*DD*	*RIG*
Median (mm)	1.4	1.2	1.2	4.0
SD (mm)	1.7	0.7	0.7	2.2
Max (mm)	6.3	3.8	3.1	10
Min (mm)	0.4	0.2	0.2	0.3
		*50%–70%*		
*Phases*	*OF*	*DM*	*DD*	*RIG*
Median (mm)	0.7	1.1	0.6	2.1
SD (mm)	0.7	0.5	0.4	1.1
Max (mm)	3.3	2.7	2.1	4.3
Min (mm)	0.1	0.1	0.1	0.3

**Figure 3:**
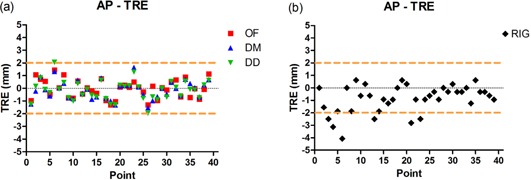
Target registration errors (TRE) of the three DIR methods (a) in the anterior–posterior (AP) direction with 0%–50% registration (the maximum TRE for all three DIRs was less than two voxels, marked with the dashed lines); the distance in AP (b) between the landmarks without any registration.

**Figure 4:**
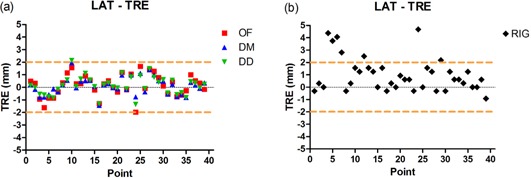
Target registration error in the lateral direction (a) with 0%–50% registration; all three DIR methods performed similarly and had target registration error within two voxels, shown by the two dashed lines. The distance in lateral (b) between the landmarks without any registration.

**Figure 5:**
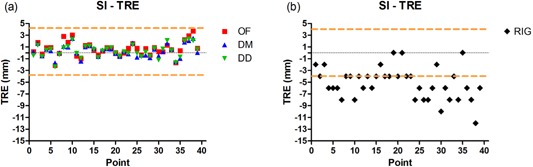
Target registration error in the superior–inferior (SI) direction (a), with 0%–50% registration; the target registration errors were within two voxels, shown by the two dashed lines. The distance in SI direction (b) between the landmarks without any registration.

**Figure 6:**
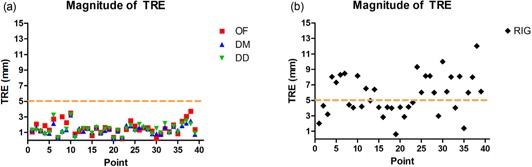
The magnitude of target registration errors (a) for the DIR methods with 0%–50% registration. The maximum TRE for all three methods was within two voxels, as denoted by the dashed line. OF mean TRE=1.6±0.9mm, DM mean TRE=1.4±0.6mm, and DD mean TRE=1.4±0.7mm. The distances (b) between the landmarks without any registration. The mean landmark distance with RIG was 5.7±2.5mm.

Figures 3 to 6 show TRE plots of the 0%–50% registration for each point, and the dashed lines in these figures represent a ± two voxel error. Figure 3(a) shows a plot of TRE in the anterior–posterior (AP) direction for the three DIR algorithms (OF, DM, and DD), and Fig. 3(b) shows the distance between the phases of landmarks in the AP with RIG only.

Figure 4(a) shows a plot of TRE in the lateral (LAT) direction for the three DIR algorithms, and Fig. 4(b) shows the distance between the phases of landmarks in the LAT without any registration. Figure 5(a) shows a plot of TRE in the superior–inferior (SI) direction for the three DIR algorithms, and Fig. 5(b), shows the distance between the phases of landmarks in the SI without any registration. Figure 6(a) shows the magnitude of the TRE for DIR and Fig. 6(b) shows the magnitude of landmark distance without DIR.

**Figure 7:**
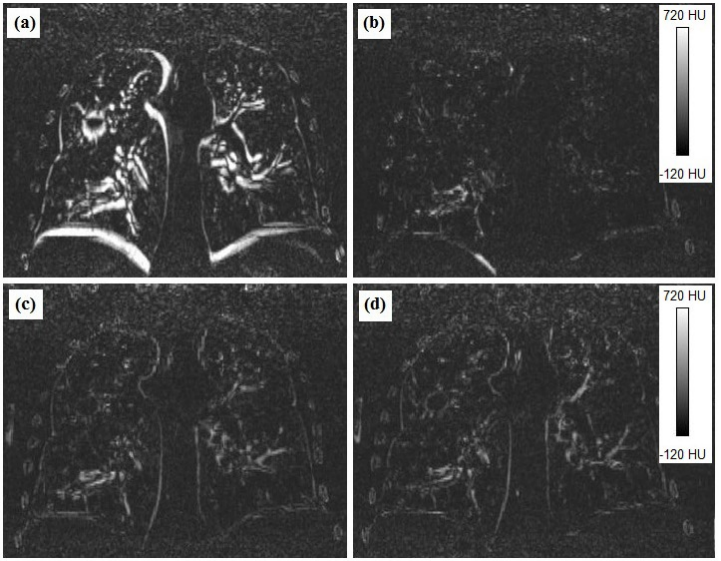
Image differences between the deformed and the target images for: (a) RIG, (b) optical flow, (c) diffeomorphic morphons, and (d) diffeomorphic demons.

**Figure 8:**
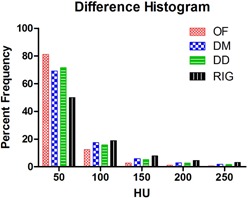
Histogram of the differences between the deformed and the target images. The histogram shows the differences in HU between the images as a percentage of the total number of voxels encompassed by the region of interest. The RIG shows the HU difference between the moving and the target images.

Image difference is another validation method for the DIR algorithms to determine the degree of accuracy of deformable registration. Figure 7 shows a representative slice of the image difference for the three DIR algorithms and the differences between the two image phases with RIG only. For the OF algorithm, 81% of voxels were within a difference of 50 HU and 93% of the voxels were within 100 HU. For the DM algorithm, 69% of voxels were within 50 HU and 87% within 100 HU. For the DD algorithm, 71% of the voxels were within 50 HU and 87% within a difference of 100 HU. The image differences with RIG only between the moving and the target images were 50% within 50 HU and 69% within 100 HU. Figure 8 displays a histogram of the differences between the target and the deformed images as a percentage of the total voxels for all the algorithms, as well as the difference between the target and the moving images without a DIR to set a baseline. Overall differences between the DIR algorithms are small.

## IV. DISCUSSION

Various groups have used different models to validate image registration algorithms.^(^ ^25^ ^,^ ^26^ ^,^ ^31^ ^,^ ^32^ ^)^ Janssens et al.^(^ ^25^ ^)^ used metallic fiducial markers implanted inside a homogeneous phantom to evaluate deformable image registration with morphons and demons. There are several issues with using phantoms for evaluating DIR algorithms. Phantoms in general are not deformed in the same manner as a normal patient's anatomy; they also deform reproducibly, thus producing superior 4D CT image sets without the variability of actual patient breathing. The deformations of the phantom may also be more predictable and smoothly varying. Phantoms are a good method for testing DIR, but results may not be directly related to 4D CT imaging of patients. Additionally, if phantoms are deformed using mathematical models, then the CT artifacts, noise, and quantum effects may be exactly the same in the image pairs, which would not affect the registration quality. However, for registrations between different phases of 4D CT images, noise and image artifacts are not the same across images and, therefore, degrade the quality of registration.

In a different study, Janssens et al.^(^ ^20^ ^)^ reported similar results to ours when validating DM and DD using the POPI model. They found that the mean target registration error for DM was 0.9±0.5mm and a maximum of 2.8 mm, the mean target registration error for DD was 1.0±0.5mm and a maximum of 2.8 mm. The reason for the differences between our results may be due to the differences in image resolution, as the voxel size for their study was $2\times 2\times 2\;{\text{mm}}^{3}$, or due to the difference in image quality between the two studies.

Wang et al.^(^ ^26^ ^)^ tested the demons algorithm on three different cases using a model based on real patient data. In the first case, they used CT images from a prostate patient, with voxel sizes $0.94\;\times 0.94\times 3.0\;{\text{mm}}^{3}$, and then mathematically deformed the images in a well‐defined manner. In the second case, they used CT images from a head‐and‐neck patient, with voxel sizes $0.68\;\times 0.68\times 3.0\;{\text{mm}}^{3}$, and then mathematically deformed the images; in the third case they used a deformable pelvis phantom. They found that the mean error for the first case was 0.5±1.5mm, for the second case 0.2±0.6mm, and 0.8±0.5mm for the third case. Although this is a more advanced model than phantom measurements to test the accuracy of the deformation algorithm, this method also lacks patient variability and, consequently, may overestimate the accuracy of the technique. The accuracy is deemed to be good since CT artifacts, noise, and quantum effects were exactly the same in the image pairs, which would not affect the registration quality, while for registrations between phases of 4D CT images this is not the case. The presence of different CT artifacts and noise in the images is a factor that would degrade the quality of the registration. Another source of differences between our results and results from the Wang study may be because the anatomical regions are not the same. Similarly, Zhong et al.^(^ ^32^ ^)^ validated demons algorithm using a synthesized prostate model and a CT image‐based lung model. They found that the mean displacement error in the prostate was 2.0 mm, while the mean displacement error for the lung registration was 1.3 mm.

Castillo et al.^(^ ^31^ ^)^ evaluated the OF algorithm using a large number of landmarks. They tested image registration with voxel size$5\times 5\times 5\;{\text{mm}}^{3}$ and found that the mean 3D registration error for this algorithm was 6.9±0.1mm. This method is similar to the model we used for our validation because it is based on anatomical landmarks. The voxel size used for this study is quite large and may not reflect the clinical situation; the CT voxel size of the POPI model used in our validation was $0.97\times 0.97\times 2\;{\text{mm}}^{2}$. Additionally, Castillo and colleagues tested a landmark‐based DIR algorithm and found that it performed better than the OF algorithm (error of 2.05±0.02mm). As one would expect, using landmarks to evaluate a landmark‐based algorithm may yield too optimistic results. Additionally, Castillo et al. found the error in OF was greater with DIR than RIG‐only registration for four out of the five cases they reported, while our study found the OF DIR was superior to RIG‐only registration.

In all these validation studies, the voxel size will directly determine a lower limit of accuracy that can be reached using DIR, as the anatomical landmarks are defined by a voxel. For example, the slice thickness is the limiting factor on the accuracy of the landmark points in the superior–inferior direction because a point may lie in between two slices, which would make it hard to identify by the radiologist on the two image sets. In addition, the image sets used are from a 4D CT, which may make the identification of landmarks less clear than identifying landmarks on a breath‐hold CT. Therefore, we consider the registration accuracy in this validation study for the three DIR algorithms to be acceptable, as the mean target registration errors are less than the slice thickness. This is comparable to the validation study for demon's algorithm reported by Vandemeulebroucke et al.^(^ ^24^ ^)^ Their results show a mean TRE of 1.2 mm with a standard deviation of 0.4 mm, while ours show a mean TRE of 1.4 mm with a standard deviation of 0.7 mm.

The POPI model is published and used by many groups for validation studies.^(^ ^20^ ^–^ ^22^ ^,^ ^24^ ^)^

The task of manually selecting landmarks is difficult, tedious, and time‐consuming. In addition, selecting landmarks in the end‐expiration phase is more difficult because of the change of density of the lung.^(^ ^22^ ^)^ Given the difficulties of manual landmark selection, only one set of 4D CT data is available in the model. Thus in this study, one set of data with 39 points was used in the validation.

DIR algorithms are also evaluated by studying the image difference between the deformed image and the target image. The smaller the difference between the deformed and the target images the better the DIR algorithm. HU values in the lung for the CT sets used ranged from ‐922 to near 0. This large range in values can potentially lead to large differences when comparing two images if they are not perfectly aligned. In the case of the differences between the deformed image and the target image, large differences in HU are due to the variations at interfaces within the lung. The interfaces between the blood vessels and other parts of the lung may lead to large differences in HU between image sets.

While the validation results show that all three DIR algorithms perform well, we speculate that how DIR algorithms address the imperfection of the 4D CT images may contribute to some of the inaccuracies observed.^(^ ^33^ ^)^ Each algorithm has its own strengths and weaknesses. The DM algorithm is based on matching edges and lines in a voxel, and if an image has an artifact, then that artifact would propagate throughout the process of image deformation. On the other hand, the OF and DD algorithms are intensity‐based registration algorithms, and they may deal differently with an image artifact. Additionally, the DM and DD algorithms have smoothing filters applied to their deformation fields, which may lead to additional differences in image deformation.

## V. CONCLUSIONS

We validated all three DIR algorithms for the thorax using the POPI model, which is a landmark‐based model for the lung. We also used image differences to evaluate the accuracy of DIR algorithms. For normal end‐expiration‐to‐end‐inspiration registration, all three algorithms show a maximum target registration error of less than 4 mm, or two voxels, with insignificant differences between them (p=0.37). The mean target registration error for each of the registration algorithms was less than the slice thickness of the 3D CT volumes, which suggests that the deformable registrations done by either of the algorithms are fairly accurate for the thorax region. Additionally, image differences between the target and the deformed images were comparable between the DIR algorithms.

## References

[1] Bajcsy R and Kovacic S . Multiresolution elastic matching. Computer Vision, Graphics, and Image Processing. 1989;46(1):1–21.

[2] Hajnal J V, Hill DLG, Hawkes DJ, editors. Medical Image Registration. Boca Raton, FL: CRC Press LLC; 2001.

[3] Castillo R , Castillo E , Martinez J , Guerrero T . Ventilation from four‐dimensional computed tomography: density versus Jacobian methods. Phys Med Biol. 2010;55(16):4661–85. 20671351 10.1088/0031-9155/55/16/004

[4] Guerrero T , Sanders K , Castillo E , et al. Dynamic ventilation imaging from four‐dimensional computed tomography. Phys Med Biol. 2006;51(4):777–91. 16467578 10.1088/0031-9155/51/4/002

[5] Guerrero T , Sanders K , Noyola‐Martinez J , et al. Quantification of regional ventilation from treatment planning CT. Int J Radiat Oncol Biol Phys. 2005;62(3):630–34. 15936537 10.1016/j.ijrobp.2005.03.023

[6] Reinhardt JM , Christensen GE , Hoffman EA , Ding K , Cao K . Registration‐derived estimates of local lung expansion as surrogates for regional ventilation. Inf Process Med Imaging. 2007;20:763–74. 17633746 10.1007/978-3-540-73273-0_63

[7] Yamamoto T , Kabus S , Klinder T , et al. Investigation of four‐dimensional computed tomography‐based pulmonary ventilation imaging in patients with emphysematous lung regions. Phys Med Biol. 2011;56(7):2279–98. 21411868 10.1088/0031-9155/56/7/023

[8] Yamamoto T , Kabus S , von Berg J , Lorenz C , Keall PJ . Impact of four‐dimensional ct‐derived pulmonary ventilation images on radiotherapy treatment planning for lung cancer [abstract]. Int J Radiat Oncol Biol Phys. 2009;75:S443. 10.1016/j.ijrobp.2010.02.008 20646852

[9] Yamamoto T , Kabus S , von Berg J , et al. Four‐dimensional computed tomography‐based pulmonary ventilation imaging for adaptive functional guidance in radiotherapy. J Thoracic Oncol. 2009;4:S959–S960.

[10] Zhang G , Dilling TJ , Stevens CW , Forster KM . Functional lung imaging in thoracic cancer radiotherapy. Cancer Control. 2008;15(2):112–19. 18376378 10.1177/107327480801500203

[11] Zhang G , Huang TC , Dilling TJ , Stevens C , Forster KM . Derivation of high‐resolution pulmonary ventilation using local volume change in four‐dimensional ct data. In: Proceedings of the World Congress on Medical Physics and Biomedical Engineering, Sept 7–12, 2009, Munch, Germany. Paris, France: IFMBE; 2009.

[12] Beauchemin SS and Barron JL . The computation of optical flow. ACM Comput Surv. 1995;27(3):433–66.

[13] Horn BKP and, Schunck BG . Determining optical flow. Artificial Intelligence 1981;17(1‐3):185–203.

[14] Zhang G , Huang TC , Guerrero T , et al. Use of three‐dimensional (3D) optical flow method in mapping 3D anatomic structure and tumor contours across four‐dimensional computed tomography data. J Appl Clin Med Phys. 2008;9(1):59–69. 10.1120/jacmp.v9i1.2738 PMC5721534 18449166

[15] Zhang GG , Huang TC , Forster KM , et al. Dose mapping: validation in 4D dosimetry with measurements and application in radiotherapy follow‐up evaluation. Comput Methods Programs Biomed. 2008;90(1):25–37. 18178288 10.1016/j.cmpb.2007.11.015

[16] Pennec X , Cachier P , Ayache N . Understanding the demon's algorithm: 3D non‐rigid registration by gradient descent. In Taylor C, Colchester A, editors. Medical Image Computing and Computer‐Assisted Intervention –MICCAI'99. Vol 1679 Berlin / Heidelberg: Springer; 1999 p. 597–605.

[17] Thirion JP . Image matching as a diffusion process: an analogy with Maxwell's demons. Med Image Anal. 1998;2(3):243–60. 9873902 10.1016/s1361-8415(98)80022-4

[18] Vercauteren T , Pennec X , Perchant A , Ayache N . Diffeomorphic demons: efficient non‐parametric image registration. Neuroimage. 2009;45(Suppl 1):S61–S72. 19041946 10.1016/j.neuroimage.2008.10.040

[19] Wrangsjo A , Pettersson J , Knutsson H . Non‐rigid registration using morphons. Image Analysis. 2005;3540:501–10.

[20] Janssens G , Jacques L , Orban de Xivry J , Geets X , Macq B . Diffeomorphic registration of images with variable contrast enhancement. Int J Biomed Imaging. 2011;2011:891–585. 10.1155/2011/891585 PMC3005125 21197460

[21] Kabus S , Klinder T , Murphy K , van Ginneken B , Lorenz C , Pluim JPW . Evaluation of 4D‐CT lung registration. Proceedings of the 12th International Conference on Medical Image Computing and Computer‐Assisted Intervention: Part I. London, UK: Springer‐Verlag; 2009. 10.1007/978-3-642-04268-3_92 20426055

[22] Sarrut D , Boldea V , Miguet S , Ginestet C . Simulation of four‐dimensional CT images from deformable registration between inhale and exhale breath‐hold CT scans. Med Phys. 2006;33(3):605–17. 16878564 10.1118/1.2161409

[23] Vaman C , Staub D , Williamson J , Murphy MJ . A method to map errors in the deformable registration of 4DCT images. Med Phys. 2010;37(11):5765–76. 21158288 10.1118/1.3488983 PMC2973991

[24] Vandemeulebroucke J , Sarrut D , Clarysse P . The POPI‐model, a point‐validated pixel‐based breathing thorax model. In: Proceedings of the XVth International Conference on the Use of Computers in Radiation Therapy (ICCR), Toronto, Canada 2007.

[25] Janssens G , de Xivry JO , Fekkes S , et al. Evaluation of nonrigid registration models for interfraction dose accumulation in radiotherapy. Med Phys. 2009;36(9):4268–76. 19810501 10.1118/1.3194750

[26] Wang H , Dong L , O'Daniel J , et al. Validation of an accelerated ‘demons’ algorithm for deformable image registration in radiation therapy. Phys Med Biol. 2005;50(12):2887–905. 15930609 10.1088/0031-9155/50/12/011

[27] Guerrero T , Zhang G , Huang TC , Lin KP . Intrathoracic tumour motion estimation from CT imaging using the 3D optical flow method. Phys Med Biol. 2004;49(17):4147–61. 15470929 10.1088/0031-9155/49/17/022

[28] Kalviainen H , Parkkinen J , Kaarna A , et al. Non‐rigid registration using morphons. In: Image Analysis, Vol. 3540 Berlin / Heidelberg: Springer; 2005 p. 501–10.

[29] Gu XJ , Pan H , Liang Y , et al. Implementation and evaluation of various demons deformable image registration algorithms on a GPU. Phys Med Biol. 2010;55(1):207–19. 20009197 10.1088/0031-9155/55/1/012 PMC7540904

[30] Keall PJ , Starkschall G , Shukla H , et al. Acquiring 4D thoracic CT scans using a multislice helical method. Phys Med Biol. 2004;49(10):2053–67. 15214541 10.1088/0031-9155/49/10/015

[31] Castillo R , Castillo E , Guerra R , et al. A framework for evaluation of deformable image registration spatial accuracy using large landmark point sets. Phys Med Biol. 2009;54(7):1849–70. 19265208 10.1088/0031-9155/54/7/001

[32] Zhong HL , Kim J , Chetty IJ . Analysis of deformable image registration accuracy using computational modeling. Med Phys. 2010;37(3):970–79. 20384233 10.1118/1.3302141 PMC3188658

[33] Yamamoto T , Langner U , Loo BW, Jr. , Shen J , Keall PJ . Retrospective analysis of artifacts in four‐dimensional CT images of 50 abdominal and thoracic radiotherapy patients. Int J Radiat Oncol Biol Phys. 2008;72(4):1250–58. 18823717 10.1016/j.ijrobp.2008.06.1937 PMC2583232

